# Mode of Obstetric Delivery in Kidney and Liver Transplant Recipients and Associated Maternal, Neonatal, and Graft Morbidity During 5 Decades of Clinical Practice

**DOI:** 10.1001/jamanetworkopen.2021.27378

**Published:** 2021-10-04

**Authors:** Ophelia Yin, Aneesh Kallapur, Lisa Coscia, Lorna Kwan, Megha Tandel, Serb an Constantinescu, Michael J. Moritz, Yalda Afshar

**Affiliations:** 1 Division of Maternal Fetal Medicine, Department of Obstetrics and Gynecology, David Geffen School of Medicine, University of California, Los Angeles; 2 Transplant Pregnancy Registry International, Gift of Life Institute, Philadelphia, Pennsylvania; 3 Department of Urology, David Geffen School of Medicine, University of California, Los Angeles; 4 Section of Nephrology, Department of Medicine, Lewis Katz School of Medicine, Temple University, Philadelphia, Pennsylvania; 5 Department of Surgery, Lehigh Valley Health Network, Allentown, Pennsylvania; 6 Department of Surgery, Morsani College of Medicine, Tampa, Florida

## Abstract

**Importance:**

Rates of cesarean delivery (CD) are increased among transplant recipients. There is a need to define the indications for CD and associated outcomes among transplant recipients to determine the safest mode of obstetric delivery.

**Objective:**

To evaluate the association of mode of obstetrical delivery with maternal and neonatal morbidity among pregnant women who have received a kidney or liver transplant.

**Design, Setting, and Participants:**

This registry-based retrospective cohort study used data from the Transplant Pregnancy Registry International, which has recruited participants since 1991 from 289 diverse academic and community settings, mainly in North America. Eligible participants were recipients of a kidney or liver transplant who were aged 18 years or older at the time of a live birth at or later than 20 weeks’ gestational age and who delivered between 1968 and 2019. The data were analyzed from April 30, 2020, to April 16, 2021.

**Exposures:**

Scheduled CD, a trial of labor resulting in CD (TOL-CD), or a TOL resulting in vaginal delivery (TOL-VD).

**Main Outcomes and Measures:**

The primary outcomes were severe maternal morbidity and neonatal composite morbidity. Multivariate regression was conducted to calculate odds ratios (ORs) or β values and 95% CIs with adjustment for differences in maternal comorbidities and gestational age at delivery. Nonmedical indications for CD are those not associated with decreased morbidity or mortality in the obstetric literature.

**Results:**

This study included 1865 women, of whom 1435 were kidney transplant recipients and 430 were liver transplant recipients. The age range of the participants was 18 to 48 years; the median body mass index among the participants was in the normal range, and the median transplant-to-conception interval was more than 2 years. Compared with a scheduled CD, a TOL was not associated with increased severe maternal morbidity among kidney transplant recipients (TOL-CD: adjusted odds ratio [aOR], 1.80 [95% CI, 0.77-4.22]; TOL-VD: aOR, 1.22 [95% CI, 0.57-2.62]) (for liver transplant recipients, the numbers were too small for multivariate modeling). In the adjusted model, a TOL was associated with a decrease in neonatal composite morbidity among kidney transplant recipients who underwent TOL-CD (aOR, 0.52; 95% CI, 0.32-0.82) and TOL-VD (aOR, 0.36; 95% CI, 0.24-0.53) and liver transplant recipients who underwent TOL-VD (aOR, 0.41; 95% CI, 0.19-0.87) but not for TOL-CD (aOR, 0.58; 95% CI, 0.21-1.61). The main factors associated with CD after labor were placental abruption (aOR, 12.96; 95% CI, 2.85-59.07) and pregestational diabetes (aOR 5.44; 95% CI, 2.54-11.68). The rate of CD was 51.6% (741 of 1435) among kidney transplant recipients and 41.4% (178 of 430) among liver transplant recipients. In total, 229 of 459 kidney transplant recipients (49.9%) and 50 of 105 liver transplant recipients (47.6%) had scheduled CDs performed for either a nonmedical indication or a repeated indication, although women with these indications are candidates for a TOL.

**Conclusions and Relevance:**

In this cohort study, TOL vs a scheduled CD was associated with improved neonatal outcomes among kidney and transplant recipients and not with increased severe maternal morbidity among kidney transplant recipients. These findings may be used to facilitate multidisciplinary decisions regarding the mode of obstetrical delivery.

## Key Points

**Question:**

Are there differences in maternal and neonatal morbidity associated with the mode of obstetrical delivery among kidney and liver transplant recipients?

**Findings:**

In this cohort study of 1865 women, including 1435 kidney and 430 liver transplant recipients, a trial of labor was not associated with severe maternal morbidity in kidney recipients. Trial of labor was associated with decreased neonatal morbidity in both kidney and liver recipients compared with a scheduled cesarean delivery.

**Meaning:**

The findings suggest that, for kidney and liver transplant recipients, a trial of labor instead of scheduled cesarean delivery is associated with improved neonatal outcomes without compromising maternal health.

## Teaser

This cohort study used data from the Transplant Pregnancy Registry International to assess associations between the mode of obstetrical delivery after kidney or liver transplant and maternal and neonatal morbidity.

## Introduction

Cesarean delivery (CD), the most common operating room procedure in the US, accounted for 31.9% of obstetrical deliveries in 2018,^1^ increased from 20.7% in 1996.^2^ There is substantial maternal and neonatal morbidity associated with CD, including increased risks for maternal hemorrhage requiring transfusion or hysterectomy, infection, venous thromboembolism, abnormal placentation, and uterine rupture in subsequent pregnancies.^3,4^ Neonatal complications include respiratory morbidity and neonatal intensive care unit (NICU) admission.^5^ Recognizing these risks, national organizations have championed the importance of decreasing the rate of nonmedically indicated CDs, such as those prompted by patient preference or limited evidence, because they have not been shown to be associated with decreased maternal or neonatal morbidity or mortality.^5,6^ Medical indications for a CD include fetal malpresentation, history of a uterine scar in the contractile tissue of the uterus (such as from a classical CD or myomectomy), abnormal placentation such as placenta accreta, abnormal labor course based on contemporary labor curves, nonreassuring fetal heart tracing, complicated delivery with twins or higher-order multiples, and suspected macrosomia with diabetes.^6^ Other than these contraindications to labor, obstetric guidelines recommend offering patients, including those who have had a previous CD, a trial of labor (TOL) to avoid a CD in appropriate candidates.^6^

Approximately 15 000 women underwent an organ transplant in 2020 in the US, and 35% of female recipients were in the reproductive age group.^7^ Pregnancies after kidney and liver transplant represent a small proportion of pregnancies overall, but women who have received these transplants have a disproportionately increased rate of CD (62.6% among kidney recipients and 44.6% among liver recipients).^8,9^ Among patients, obstetricians, and transplant teams, there is a continued practice of nonmedically indicated CD, although the true rates are still unknown.^10,11,12,13^ Expert consensus states that CD in transplant recipients should be reserved for obstetric medical indications,^14^ but these recommendations are similarly based on scant data. To our knowledge, there is no robust evidence to guide the safest mode of delivery (MOD) for pregnancies after organ transplant.

Our aim was to evaluate differences in pregnancy-related morbidity associated with MOD after kidney or liver transplant. Our hypothesis was that a scheduled CD (SCD) would be associated with an increase in maternal morbidity without graft or neonatal benefit and that a TOL would optimize overall outcomes associated with delivery.

## Methods

### Study Design, Participants, Setting, and Data Collection

This cohort study used retrospective registry data abstracted from the Transplant Pregnancy Registry International (TPRI), which has recruited participants since 1991. Data from pregnancies delivered between 1968 and 2019 were analyzed from April 30, 2020, to April 16, 2021. The study followed the Strengthening the Reporting of Observational Studies in Epidemiology (STROBE) reporting guideline. The TPRI and its associated studies have been approved by the Advarra institutional review board, and all participants provided either oral or written informed consent. To our knowledge, the TPRI is the longest running voluntary registry of pregnancies after transplant, and it enrolls participants from 289 diverse academic and community centers and hospitals, mainly in North America.

For the TPRI, trained research coordinators and physicians follow up participants within 1 month after delivery and then every 1 to 2 years. Pregnancy information is obtained through patient interviews and medical record review. Race and ethnicity are self-reported, with options defined by the TPRI investigators, and were included in this study based on the association of race and ethnicity with obstetric morbidity in a prior study.^15^

Eligible participants were recipients of a kidney or liver transplant who were aged 18 years or older at the time of a live birth at or later than 20 weeks’ gestational age. Women with multiple gestations and fetal anomalies were included. Participants were excluded if the MOD was unknown or if they had an emergency prelabor CD during an antepartum admission to the hospital because these patients had no alternative MOD. If a participant had more than 1 pregnancy, each pregnancy was treated as a separate encounter. All participants were followed up until at least 2 years post partum to capture data on short-term graft loss.

### Exposures and Outcomes

Three cohorts were identified for statistical analysis: (1) SCD for any indication, (2) TOL resulting in CD (TOL-CD), and (3) TOL resulting in vaginal delivery (TOL-VD), including both spontaneous and operative vaginal deliveries such as those using a vacuum or forceps. Each pregnancy and delivery indication was reviewed by one of us (O.Y.) for accuracy.

The primary outcomes of this study were (1) severe maternal morbidity, defined as 1 or more of the 21 indicators identified by the Centers for Disease Control and Prevention (eg, eclampsia, sepsis, blood products transfusion, and hysterectomy) occurring intrapartum or within 6 weeks post partum,^16^ and (2) neonatal composite morbidity, defined as 1 or more of the Maternal-Fetal Medicine Units adverse outcomes defined by the National Institute of Child Health and Human Development, National Institutes of Health, including intraventricular hemorrhage, hypoxic ischemic encephalopathy, seizure, sepsis, necrotizing enterocolitis, bronchopulmonary dysplasia, persistent pulmonary hypertension, respiratory distress syndrome, fracture, brachial plexus injury, cardiopulmonary resuscitation, or perinatal death.^17,18^

Secondary outcomes included the following maternal outcomes: postpartum hemorrhage; intra-amniotic infection, defined as uterine infection intrapartum or within 24 hours post partum; surgical site infection, defined as any perineal, uterine, or wound infection requiring antibiotics more than 24 hours after delivery; postpartum readmission to the hospital; and graft loss within 2 years of delivery. The following neonatal outcomes were also assessed: Apgar score less than 7 at 1 minute, Apgar score less than 7 at 5 minutes, NICU admission, and NICU length of stay.

### Statistical Analysis

**Figure 1.**
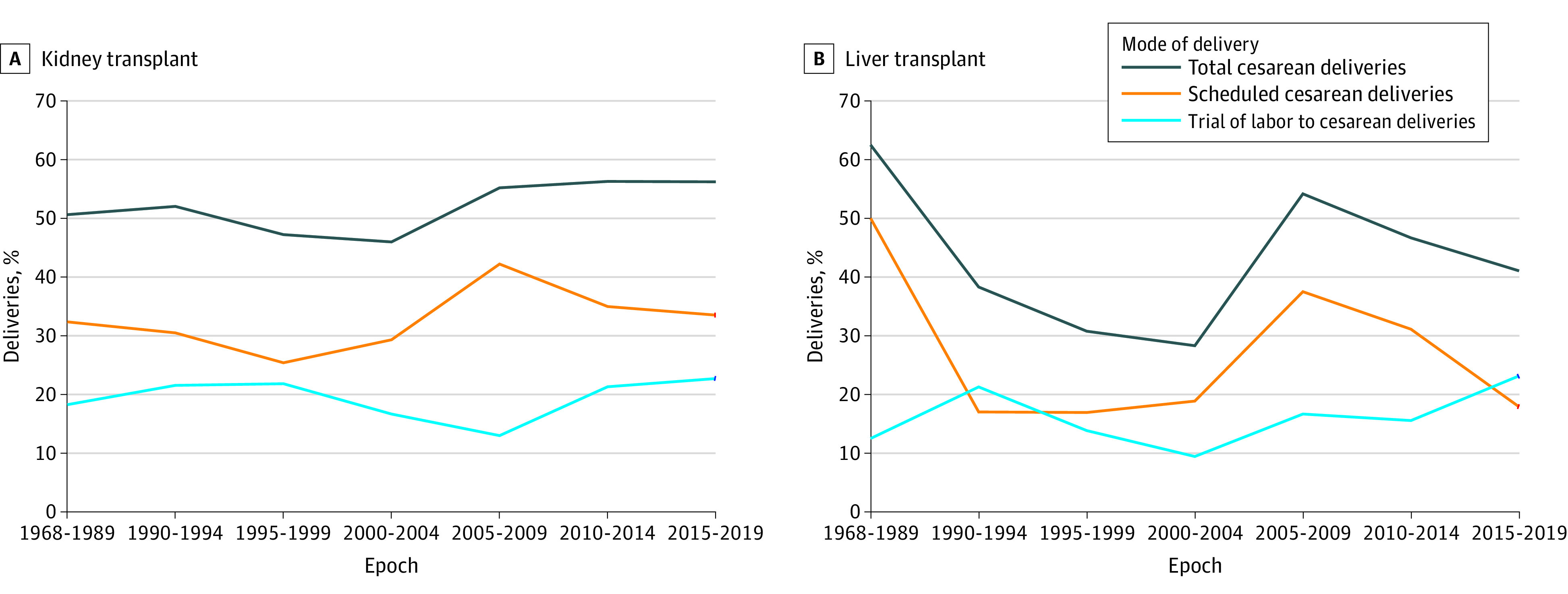
Trends in Cesarean Deliveries After Kidney and Liver Transplant by 5-Year Epochs All deliveries included both cesarean and vaginal deliveries. Owing to small numbers, the first epoch included 1968 to 1989.

The study population was stratified into recipients of a kidney or a liver transplant to understand morbidity by organ. We calculated the CD rates in 5-year epochs, shown as a graph spanning the study period (Figure 1). Owing to small numbers, the first epoch included 1968 to 1989. We also compared maternal and neonatal characteristics and outcomes by the 3 MODs with χ^2^ and *t* tests (or Wilcoxon rank-sum tests as appropriate). We compared indications for MOD by organ.

Multivariate regression was conducted to calculate adjusted odds ratios (aORs) or adjusted β values and 95% CIs. The independent variable was the MOD; patients undergoing an SCD were used as the reference group. Covariates chosen a priori to include in the models were hypertension, pregestational diabetes, maternal age, body mass index (BMI; calculated as weight in kilograms divided by height in meters squared), race and ethnicity, multiple gestation, nulliparity, and in vitro fertilization.^19,20,21,22,23,24,25,26^ Models were also adjusted for a short transplant-to-conception interval (<2 years) given its association with neonatal mortality^8^ and graft rejection^27^ and for year of delivery after 2000. The transition at 2000 was hypothesized to be the most sensitive to historical trends for 2 reasons. The first was the decrease in use of TOL after a CD beginning in 1996 owing to concerns about uterine rupture.^28^ This decrease led to increased rates of SCDs that have persisted. The second reason was key advents in transplant therapeutics, including tacrolimus in 1994^29^ and the approval and recognition of teratogenicity for mycophenolate mofetil in pregnancy in 1995 and 2007, respectively. For neonatal outcomes, the models were further controlled for gestational age at delivery. Given that BMI was missing for 424 (29.5%) of the kidney recipients and 77 (17.9%) of the liver recipients, a sensitivity analysis was conducted on the final models. If the results were not different by more than 10%, the final models did not include BMI to preserve the full sample size for statistical analysis.

We analyzed the subset of women who had a TOL to compare characteristics associated with TOL-CD vs TOL-VD, and the resulting multivariable model was adjusted for factors associated with CD in the nontransplant population.^30,31,32^ Because Bishop scores were not available, induction of labor was used as a proxy for an unfavorable cervix at the time of admission to the hospital. All tests were 2-sided with significance set at *P* < .05. All analyses were performed using SAS, version 9.4 (SAS Institute Inc).

## Results

This study included 1865 women, of whom 1435 were kidney transplant recipients and 430 were liver transplant recipients. The age range of the participants was 18 to 48 years. For all 3 MODs among recipients of either organ, there was a similar distribution of participants by year of delivery and race and ethnicity. The median BMI among the participants was in the normal range, and the median transplant-to-conception interval was more than 2 years. A total of 1536 participants (82.4%) received prenatal care, and 614 (32.9%) delivered at their transplant center (Table 1).

**Table 1.** Maternal and Neonatal Characteristics by Mode of Delivery Among Kidney and Liver Transplant Recipients^a^

**Characteristic**	**Kidney transplant**			**Liver transplant**		
	**SCD**	**TOL-CD**	**TOL-VD**	**SCD**	**TOL-CD**	**TOL-VD**
**Maternal**						
Mothers, No.	459	282	694	105	73	252
Age, mean (SD), y	31.0 (6.6)^b^	29.4 (5.1)	30.1 (5.0)	29.9 (5.4)	29.4 (5.8)	28.8 (5.2)
BMI, median (IQR)	23.8 (21.0-28.6)^b^	25.1 (21.6-30.7)	23.0 (20.4-26.6)	23.8 (21.6-29.2)^b^	24.3 (22.0-28.4)	22.5 (20.3-26.4)
Nulliparous	224 (48.8)^b^	195 (69.1)	401 (57.8)	37 (33.3)^b^	55 (75.3)	122 (19.0)
Multiple gestations	33 (7.2)^b^	4 (1.4)	16 (2.3)	10 (9.5)^b^	0	4 (1.6)
In vitro fertilization	19 (4.1)^c^	5 (1.8)	11 (1.6)	9 (8.6)^c^	1 (1.4)	4 (1.6)
Year of delivery						
1968-1999	217 (47.3)	152 (53.9)	370 (53.3)	23 (21.9)	20 (27.4)	77 (30.1)
2000-2019	242 (52.7)	130 (46.1)	324 (46.7)	82 (78.1)	53 (72.6)	175 (69.4)
Race and ethnicity						
Asian or Asian Pacific Islander	24 (5.2)	17 (6.0)	48 (6.9)	5 (4.8)	4 (5.5)	9 (3.6)
Black	28 (6.1)	20 (7.1)	41 (5.9)	6 (5.7)	11 (15.1)	16 (6.3)
White	324 (70.6)	198 (70.2)	499 (71.9)	72 (68.6)	46 (63.0)	184 (73.0)
Other^d^	48 (10.5)	20 (7.1)	54 (7.8)	17 (16.2)	6 (8.2)	33 (13.1)
Unknown	35 (7.6)	27 (9.6)	52 (7.5)	5 (4.8)	6 (8.2)	10 (4.0)
Prenatal care	380 (82.8)	234 (83.0)	578 (83.2)	90 (85.7)	54 (74.0)	200 (79.4)
Transplant center delivery	137 (29.8)	107 (37.9)	240 (34.6)	28 (26.7)	25 (34.2)	77 (30.6)
Transplant-to-conception interval, median (IQR), y	4.9 (2.5-8.2)^c^	3.9 (2.1-6.6)	4.4 (2.3-7.6)	7.4 (3.6-15.3)	6.4 (2.1-12.5)	6.2 (3.0-11.7)
Chronic hypertension	121 (26.4)	72 (25.5)	198 (28.5)	17 (16.2)	10 (13.7)	26 (10.3)
Hypertensive disorder during pregnancy	198 (43.1)^b^	137 (48.6)	224 (32.3)	30 (28.6)	24 (32.9)	62 (24.6)
Diabetes						
Gestational	26 (5.7)	11 (3.9)	22 (3.2)	9 (8.6)	3 (4.1)	11 (4.4)
Pregestational	26 (5.7)^b^	23 (8.2)	13 (1.9)	4 (3.8)	4 (5.5)	5 (2.0)
Placental abruption	8 (1.7)^c^	7 (2.5)	3 (0.4)	3 (2.9)	2 (2.7)	5 (2.0)
**Neonatal**						
Neonates, No.	494	285	707	113	73	254
Sex						
Female	219 (44.3)	135 (47.4)	351 (49.6)	57 (50.4)	35 (47.9)	127 (50.0)
Male	275 (55.7)	150 (52.6)	356 (50.4)	56 (49.6)	38 (52.1)	125 (49.2)
Gestational age, median (IQR), wk	36 (33.0-37.9)^b^	37 (35.0-38.3)	37 (35.0-38.1)	37 (34.5-38.0)^b^	38 (35.0-39.0)	38 (36.0-39.4)
Birth weight, median (IQR), g	2495 (1860-2977)^b^	2750 (2084-3118)	2778 (2325-3141)	2736 (1973-3133)^b^	2892 (2353-3260)	2977 (2551-3345)
Weight percentile, median (IQR)^e^	35.2 (12.8-62.3)	30.2 (16.1-64.1)	32.8 (16.1-64.1)	38.4 (16.1-60.9)	30.2 (10.9-62.2)	39.3 (16.9-64.1)
Congenital anomalies	20 (4.1)	14 (4.9)	28 (4.0)	20 (4.1)	14 (4.9)	28 (4.0)

Abbreviations: BMI, body mass index (calculated as weight in kilograms divided by height in meters squared); CD, cesarean delivery; SCD, scheduled CD; TOL, trial of labor; VD, vaginal delivery.

^a^ Data are presented as number (percentage) of mothers or neonates unless otherwise indicated. Variables with more than 5% of data missing included BMI (kidney, 424 [29.5%]; liver, 77 [17.9%]), prenatal care (kidney, 117 [8.2%]; liver, 39 [9.1%]), and delivery at a transplant center (kidney, 359 [25.0%]; liver, 34 [7.9%]).

^b^ *P* ≤ .01.

^c^ *P* ≤ .05.

^d^ Other was an option provided by the Transplant Pregnancy Registry International investigators for self-reported race and ethnicity.

^e^ Fenton percentile for preterm neonates and World Health Organization percentile for term neonates.

**Figure 2.**
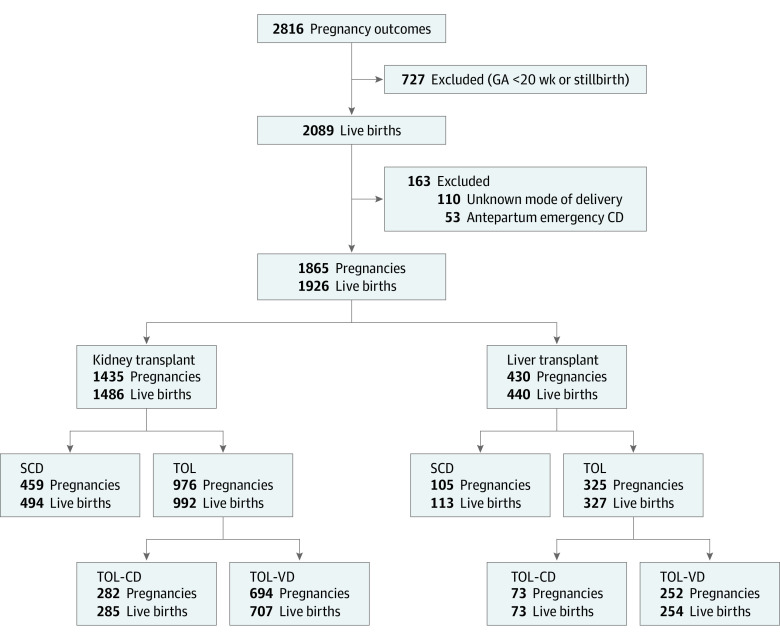
Kidney and Liver Transplant Recipients With a Live Birth Between 1968 and 2019 Who Were Enrolled in the Transplant Pregnancy Registry International CD indicates cesarean delivery; GA, gestational age; SCD, scheduled CD; TOL, trial of labor; and VD, vaginal delivery.

In the entire TPRI data set, there were 2816 total pregnancy outcomes among kidney and liver organ transplant recipients from 1968 to 2019. After exclusion of 727 patients with birth at a gestational age less than 20 weeks or a stillbirth, 2089 live births remained. We further excluded 110 live births with no known MOD and 53 emergency antepartum CDs. Our analytic cohort comprised 1865 pregnancies resulting in 1926 live births. Of these, 1435 pregnancies and 1486 live births were among kidney recipients and 430 pregnancies and 440 live births were among liver recipients (Figure 2).

### Maternal and Neonatal Characteristics According to MOD

The rate of CD was 51.6% (741 of 1435) among kidney transplant recipients and 41.4% (178 of 430) among liver transplant recipients. Among the 1435 kidney transplant recipients, 459 (32.0%) underwent SCD and 976 (68.0%) underwent a TOL. Of these, 282 (28.9%) resulted in TOL-CD and 694 (71.1%) in TOL-VD. Among the 430 liver transplant recipients, 105 (24.4%) underwent SCD and 325 (75.6%) underwent TOL, of which 73 (22.5%) resulted in TOL-CD and 252 (77.5%) in TOL-VD. Among kidney and liver recipients, those in the SCD group were more likely to have multiple gestations and a history of in vitro fertilization, and those in the TOL-CD group were more likely to have a higher BMI and nulliparity (Table 1). Among the kidney recipients, compared with those who underwent TOL-VD, those who underwent CD had higher rates of hypertensive disorders during pregnancy, pregestational diabetes, and placental abruption. Kidney recipients who underwent TOL-CD had the shortest transplant-to-conception interval (median, 3.9 years [IQR, 2.1-6.6 years]). There was no substantial difference in the numbers of women with other antepartum comorbidities, including prenatal infection, antepartum admission to the hospital, preterm labor, or anticoagulation, by MOD (eTable 1 in the Supplement).

Neonates who were delivered by SCD were born at an earlier median gestational age, although still near term, and had lower median birth weights (Table 1). Congenital anomalies affected 125 neonates (6.4%) among all MODs, concordant with the reported rate of anomalies in pregnancies among women who have not received an organ transplant.^33^

### Rates of CD During 5 Decades

Rates of CD showed an increasing trend over time. The total rate of CD was 46.8% (118 of 252 patients) to 52.0% (128 of 246) before 2000, with rates of SCD of 25.0% (63 of 252) to 32.9% (79 of 241). Between 2005 and 2009, there was a peak reflecting a total rate of CD of 55.2% (85 of 154 patients) and a rate of SCD of 42.2% (65 of 154). In the most recent period, from 2015 to 2019, a CD rate of 56.2% (104 of 185 patients) and an SCD rate of 34.1% (63 of 185) reflected a sustained increase in CDs among kidney transplant recipients compared with the 1990s. There was greater variation in rates of CD among liver transplant recipients, with smaller numbers in each epoch. Liver transplant recipients similarly had a peak in the rate of SCD from 2005 to 2009, with a total rate of CD of 54.2% (39 of 72 patients) and a rate of SCD of 37.5% (27 of 72). There was a decrease in the rate of CD to 41.1% (39 of 95 patients) and in the rate of SCD to 17.9% (17 of 95) between 2015 and 2019 among liver transplant recipients. The rates of TOL-CD were 13.0% (20 of 154 patients) to 23.2% (43 of 185) among kidney transplant recipients and 9.4% (5 of 53) to 23.2% (22 of 95) among liver transplant recipients during all epochs (Figure 2).

### Nonmedical Indications for SCD

The most common indications for an SCD among 459 kidney transplant recipients and 105 liver transplant recipients were nonmedically indicated CD (kidney: 124 [27.0%]; liver: 21 [20.0%]) and repeated CD (kidney: 105 [22.9%]; liver: 29 [27.6%]), for a combined rate of 49.9% of kidney recipients and 47.6% of liver recipients who were candidates for a TOL (eTable 2 in the Supplement). The most common indications for TOL-CD among 282 kidney transplant recipients and 73 liver transplant recipients were failure to progress in labor (kidney: 128 [45.4%]; liver: 31 [42.5%]) and nonreassuring fetal heart tracing (kidney: 99 [35.1%]; liver: 28 [38.4%]).

### Maternal and Graft Morbidity by MOD

Maternal outcomes of postpartum hemorrhage, intra-amniotic infection, and postpartum readmission to the hospital were rare for kidney and liver transplant recipients; each outcome had an incidence of less than 5% in all subgroups (eTable 3 in the Supplement). Surgical site infection was more frequent among kidney and liver transplant recipients who underwent CD compared with VD (kidney: SCD, 6 of 459 [1.3%]; TOL-CD, 5 of 282 [1.8%]; TOL-VD, 2 of 694 [0.3%]; liver: SCD, 4 of 105 [3.8%]; TOL-CD, 3 of 73 [4.1%]; TOL-VD, 0 of 252), but these increases were not significant after adjustments in the multivariate model. Among kidney transplant recipients, the primary outcome of severe maternal morbidity occurred after SCD in 13 of 459 pregnancies (2.8%), after TOL-CD in 12 of 282 (4.3%), and after TOL-VD in 18 of 694 (2.6%). Among liver recipients, severe maternal morbidity occurred after SCD in 2 of 105 pregnancies (1.9%), after TOL-CD in 3 of 73 (4.1%), and after TOL-VD in 7 of 252 (2.8%). After adjusting for maternal demographic characteristics and factors associated with morbidity, TOL was not associated with severe maternal morbidity among kidney transplant recipients (TOL-CD: aOR, 1.80 [95% CI, 0.77-4.22]; TOL-VD: aOR, 1.22 [95% CI, 0.57-2.62]); for liver transplant recipients, the numbers were too small for multivariate modeling (Table 2). In the adjusted model, TOL was not associated with the other secondary maternal outcomes. Blood products transfusion and sepsis were the most common factors associated with severe maternal morbidity (eTable 4 in the Supplement).

**Table 2.** Multivariate Analyses of Obstetric and Graft Outcomes by Mode of Delivery Among Kidney and Liver Transplant Recipients^a^

Outcome	Kidney transplant				Liver transplant			
	OR (95% CI)		aOR (95% CI)		OR (95% CI)		aOR (95% CI)	
	TOL-CD	TOL-VD	TOL-CD	TOL-VD	TOL-CD	TOL-VD	TOL-CD	TOL-VD
Maternal^b^								
Postpartum hemorrhage	1.40 (0.47-4.22)	1.72 (0.71-4.15)	1.41 (0.46-4.31)^c^	1.73 (0.71-4.23)^c^	2.93 (0.26-32.92)	4.30 (0.54-34.00)	NA	NA
Intra-amniotic infection	1.63 (0.33-8.15)	0.66 (0.13-3.28)	1.81 (0.34-9.79)^d^	0.81 (0.15-4.34)^d^	0.72 (0.06-8.04)	0.41 (0.06-2.96)	NA	NA
Surgical site infection	1.09 (0.30-3.88)	0.22 (0.04-1.09)	1.20 (0.32-4.54)^c^	0.27 (0.05-1.37)^c^	1.08 (0.23-4.99)	NO	NA	NA
Postpartum readmission	1.63 (0.33-8.15)	1.10 (0.26-4.64)	2.44 (0.45-13.21)^e^	1.40 (0.32-6.21)^e^	NO	0.41 (0.03-6.69)	NA	NA
Severe maternal morbidity	1.66 (0.73-3.74)	0.99 (0.47-2.08)	1.80 (0.77-4.22)	1.22 (0.57-2.62)	2.21 (0.36-13.55)	1.47 (0.30-7.20)	NA	NA
Graft loss within 2 y	1.02 (0.56-1.88)	0.74 (0.44-1.23)	0.92 (0.49-1.72)^f^	0.70 (0.41-1.20)^f^	0.72 (0.06-8.04)	0.83 (0.15-4.62)	0.59 (0.04-7.97)^f^	0.85 (0.14-5.37)^f^
Neonatal^g^								
Apgar score <7^h^								
At 1 min	1.53 (0.82-2.85)	1.09 (0.62-1.89)	2.51 (1.20-5.22)	1.88 (0.97-3.67)	1.03 (0.25-4.30)	0.34 (0.10-1.16)	2.49 (0.32-19.46)^i^	0.89 (0.14-5.57)^i^
At 5 min	1.43 (0.53-3.84)	0.36 (0.11-1.12)	1.79 (0.57-5.66)	0.27 (0.06-1.15)	0.73 (0.11-4.69)	NO	NA	NA
NICU								
Hospital admission	0.68 (0.48-0.96)	0.44 (0.33-0.59)	1.45 (0.94-2.24)	0.85 (0.59-1.22)	0.74 (0.37-1.45)	0.41 (0.24-0.71)	1.00 (0.41-2.45)	0.65 (0.33-1.31)
Length of stay, β (SE)	−14.2 (5.3)^j^	−13.0 (4.5)^j^	−2.7 (4.4)	−2.0 (3.7)	−21.4 (14.1)	−14.2 (11.3)	−25.0 (13.5)	−8.6 (10.4)
Neonatal composite morbidity	0.46 (0.30-0.71)	0.31 (0.22-0.44)	0.52 (0.32-0.82)	0.36 (0.24-0.53)	0.56 (0.22-1.42)	0.38 (0.19-0.77)	0.58 (0.21-1.61)	0.41 (0.19-0.87)

Abbreviations: aOR, adjusted odd ratio; BMI, body mass index (calculated as weight in kilograms divided by height in meters squared); CD, cesarean delivery; IVF, in vitro fertilization; NA, not applicable (numbers were too small for multivariate modeling); NO, outcome did not occur; NICU, neonatal intensive care unit; OR, odds ratio; TOL, trial of labor; VD, vaginal delivery.

^a^ The addition of BMI did not change the results by 10% or more; thus, it was omitted to preserve the full sample size.

^b^ For all 5 maternal outcomes, scheduled CD was the reference group and the models included year of delivery, maternal age at conception, race and ethnicity, parity, multiple gestation, IVF, hypertensive disease during pregnancy, pregestational diabetes, and transplant-to-conception interval.

^c^ The model did not include multiple gestation and pregestational diabetes owing to small numbers.

^d^ The model did not include IVF owing to small numbers.

^e^ The model did not include pregestational diabetes owing to small numbers.

^f^ The model also included prepregnancy hypertensive disease but did not include multiple gestation and IVF owing to small numbers.

^g^ For all 5 neonatal outcomes, scheduled CD was the reference group and the models included year of delivery, maternal age at conception, race and ethnicity, parity, multiple gestation, IVF, hypertensive disease during pregnancy, pregestational diabetes, transplant-to-conception interval, and gestational age.

^h^ Variables with more than 5% of the data missing included Apgar score at 1 minute (kidney, 1033 [69.5%]; liver, 361 [82.0%]) and Apgar score at 5 minutes (kidney, 1033 [69.5%]; liver, 363 [82.5%]).

^i^ The model did not include race and ethnicity, multiple gestation, IVF, and pregestational diabetes owing to small numbers.

^j^ *P* < .05, compared with scheduled CD.

Graft loss within 2 years after delivery occurred among 29 of 459 kidney recipients who underwent SCD (6.3%), 18 of 282 who underwent TOL-CD (6.4%), and 33 of 694 who underwent TOL-VD (4.8%). Graft loss among liver transplant recipients occurred among 2 of 105 who underwent SCD (1.9%), 1 of 73 who underwent TOL-CD (1.4%), and 4 of 252 who underwent TOL-VD (1.6%) (eTable 3 in the Supplement). There was no difference in short-term graft loss based on MOD in the adjusted model (Table 2). Surgical graft injury during a CD occurred in 5 of all 1865 pregnancies (0.3%), of which 2 (40.0%) occurred during SCD and 3 (60.0%) during TOL-CD.

### Neonatal Morbidity by MOD

For pregnancies after a kidney or liver transplant, NICU admission of the neonate was common, occurring in 385 (20.0%) of 1926 live births. The primary outcome of neonatal composite morbidity among neonates of kidney transplant recipients occurred for 103 of 494 in the SCD group (20.9%), 31 of 285 in the TOL-CD group (10.9%), and 53 of 707 in the TOL-VD group (7.5%). Among neonates of liver transplant recipients, neonatal composite morbidity occurred for 18 of 113 in the SCD group (15.9%), 7 of 73 in the TOL-CD group (9.6%), and 17 of 254 in the TOL-VD group (6.7%) (eTable 3 in the Supplement). Neonatal morbidity was mainly attributable to respiratory distress syndrome, with each of the other indicators occurring in less than 3% of neonates by MOD (eTable 5 in the Supplement). Among neonates of kidney transplant recipients, TOL-CD was associated with a higher risk of an Apgar score less than 7 at 1 minute (aOR, 2.51; 95% CI, 1.20-5.22) but not at 5 minutes compared with SCD. After adjusting for maternal risk factors and gestational age at delivery, TOL compared with SCD was associated with decreased odds of neonatal composite morbidity among kidney transplant recipients in the TOL-CD (aOR, 0.52; 95% CI, 0.32-0.82) and TOL-VD (aOR, 0.36; 95% CI, 0.24-0.53) groups. Among liver transplant recipients, TOL was associated with decreased neonatal morbidity for TOL-VD (aOR, 0.41; 95% CI, 0.19-0.87) but not for TOL-CD (aOR, 0.58; 95% CI, 0.21-1.61) (Table 2).

### Factors Associated With TOL-CD

Among kidney transplant recipients who had a TOL, factors associated with CD included placental abruption (aOR, 12.96; 95% CI, 2.85-59.07), pregestational diabetes (aOR, 5.44; 95% CI, 2.54-11.68), and induction of labor (aOR, 2.98; 95% CI, 2.04-4.37) (Table 3). Repeated kidney transplant (aOR, 1.79; 95% CI, 1.16-2.77) was a unique factor associated with CD. For liver transplant recipients, nulliparity (aOR, 3.45; 95% CI, 1.77-6.72), obesity (aOR, 3.83; 95% CI, 1.17-12.51), and Black race (aOR, 2.88; 95% CI, 1.06-7.85) were associated with TOL-CD.

**Table 3.** Factors Associated With Risk for TOL-CD Among Kidney and Liver Transplant Recipients

Factor	TOL-CD, aOR (95% CI)^a^	
	Kidney transplant (n = 976)	Liver transplant (n = 325)
Placental abruption	12.96 (2.85-59.07)	2.29 (0.31-16.97)
Pregestational diabetes	5.44 (2.54-11.68)	2.39 (0.47-12.06)
Induction of labor	2.98 (2.04-4.37)	1.92 (0.94-3.92)
Black race	1.49 (0.79-2.79)	2.88 (1.06-7.85)
Obesity	2.57 (1.51-4.34)	3.83 (1.17-12.51)
Gestational diabetes	1.95 (1.10-3.48)	1.36 (0.32-5.83)
Aspirin use	1.93 (1.12-3.34)	0.82 (0.26-2.61)
Hypertensive disorder	1.91 (1.28-2.85)	1.34 (0.64-2.82)
Repeated transplant	1.79 (1.16-2.77)	2.63 (0.93-7.41)
Nulliparity	1.68 (1.20-2.35)	3.45 (1.77-6.72)
Age^b^	0.96 (0.93-0.99)	1.04 (0.98-1.10)

Abbreviations: aOR, adjusted odds ratio; TOL-CD, cesarean delivery after trial of labor.

^a^ The model was adjusted for race and ethnicity, location, year of delivery, multiple gestation, chronic hypertension, preterm labor, fetal malformations, in vitro fertilization, transplant-to-conception interval, anticoagulation, gestational age, and birth weight percentile.

^b^ Each additional year.

## Discussion

The overall rate of CD in the population of patients who received an organ transplant was persistently high since the 1970s. Rates of SCD for nonmedical and repeated indications, which range from 7.3% to 67.4% in the literature,^11,12,34,35^ contribute to the overall rate of CD. In the present study, which had a larger sample size than the previous studies, the rate of avoidable CDs was closer to the higher end of these estimates, representing 279 of all 564 scheduled CDs (49.5%).

Nonmedically indicated CDs in the TPRI were prompted by physician recommendation against a TOL, patient preference for CD, and hospital policies recommending CD after a prior organ transplant. Reasons given for these recommendations, as documented in the TPRI, were fears of worsening hypertensive disease or infection, graft injury either from labor or emergent CD, low optimism for a TOL, and concern about neonatal well-being after a stressful TOL.

In this study, we found no increased maternal morbidity associated with a TOL and, in particular, no increased risk of acute kidney failure, eclampsia, pulmonary edema, stroke, or cardiovascular complication from worsening hypertensive disease during labor. Prior studies^36,37^ of pregnancies affected by severe preeclampsia similarly showed safety associated with an induction of labor. In this study, rates of intra-amniotic infection and sepsis were not increased in the TOL group. Trial of labor preceding CD may be associated with a decreased rate of surgical site infection compared with SCD in the context of immunosuppression. In this study, the increased rates of infection in univariate analysis but the lack of difference in the multivariate model was likely attributable to the inability to adjust for pregestational diabetes among kidney recipients and to the small number of liver transplant recipients. In addition, hemorrhage during a CD after a TOL can be caused by uterine atony after a prolonged labor course or uterine extensions from a thinned out, labored, lower uterine segment.^38^ Despite these factors, TOL-CD was not associated with a greater hemorrhage or transfusion risk compared with SCD.

In terms of allograft injury, experts in transplantation have stated that the allograft location is unlikely to experience damage during labor.^39^ The liver is in a distant location in the upper right quadrant of the abdomen, and the kidney is in the false pelvis at the retroperitoneal iliac fossa.^40^ Surgical injury to the allograft in an emergent CD is rare^41^ and occurred in only 0.27% of the entire study population. We found no difference in short-term graft loss based on MOD, consistent with existing literature^8,42^ supporting healthy graft function after pregnancy in individuals with immunosuppression.

In this study, most TOL candidates (72.7%) delivered by TOL-VD. Selecting ideal candidates for a TOL may maximize success and should include a discussion regarding preexisting diabetes, prior organ transplant, and favorability of the cervix during labor. Placental abruption was associated with CD; therefore, patients with this complication in the course of their labor should be carefully treated with this in mind. Black race was associated with CD in liver transplant recipients, suggesting that racism and hospital setting may contribute to the likelihood of CD during a TOL, but these results should be interpreted with caution given the low number of liver transplant recipients.

Consistent with the findings in prior obstetric literature,^5^ in this study, a TOL was associated with reduced neonatal morbidity, particularly decreased respiratory distress and need for mechanical ventilation. One prior study^11^ did not find improved neonatal outcomes associated with TOL after liver transplant, but the study was limited by a sample size of 21 deliveries (15 CDs and 6 VDs).

A TOL in kidney and liver transplant recipients has historically been seen as a higher risk delivery option within the TPRI, leading practitioners to opt for a scheduled CD. This study’s results challenged this approach, suggesting that a TOL regardless of delivery outcome is not associated with higher maternal or graft morbidity and is associated with decreased neonatal morbidity. Cesarean delivery trends in the general population suggest that inertia may occur once a practice pattern has been established. To decrease the rate of CD after organ transplant, there may need to be changes at multiple levels, including obstetric and transplant team support, delivery unit and hospital policy improvements, and national and international support. Establishing preconception and prenatal care at a patient’s transplant center may facilitate better interdisciplinary communication and management during a TOL.

### Strengths and Limitations

This study has strengths. These include the robust sample size from 5 decades; close and consistent follow-up; varying practice sites; and joint collaboration, data interpretation, and data analysis by transplantation and obstetric physicians and coordinators.

This study also has limitations. Outcomes were patient reported, with differing availability of medical records to corroborate phone interviews. Findings associated with the secondary outcomes were most subject to bias given the milder nature of the conditions. Postpartum hemorrhage and intra-amniotic infection were likely underreported because rates higher than 1% to 4% would be expected in this cohort at high risk of unfavorable outcomes.^43,44,45^ Apgar scores were limited by missing data. The outcomes of severe maternal morbidity, neonatal composite morbidity, and graft loss at 2 years post partum were expected to be less affected by recall bias owing to the severity of these events. There may have been confounders that affected MOD allocation that were not accounted for in our adjusted model. We could not adjust for placental abruption given the rarity of this event. We did not have data regarding prior CDs, which limited our ability to control for morbidity associated with repeated CDs. The findings of this study are most generalizable to pregnancies in North America and among White women.

## Conclusions

In this cohort study, TOL vs an SCD was associated with improved neonatal outcomes among kidney and liver transplant recipients and not with increased severe maternal morbidity among kidney transplant recipients. This study’s data on MODs after organ transplant during 5 decades should be critically examined within individual practice contexts and clinical actions fine tuned to ensure the best outcomes for the mother, the neonate, and the allograft.

## Notes

Supplement. **eTable 1.** Additional maternal characteristics according to mode of delivery among kidney and liver transplant recipients **eTable 2.** Primary indication for scheduled cesarean delivery (SCD) and cesarean delivery after trial of labor (TOL-CD) for pregnancies of kidney and liver transplant recipients **eTable 3.** Maternal, graft, and neonatal outcomes by mode of delivery among kidney and liver transplant recipients **eTable 4.** Individual indicators of severe maternal morbidity according to the Centers for Disease Control and Prevention **eTable 5.** Individual indicators of neonatal composite morbidity according to the NIH/NICHD
