# Error in Byline

**DOI:** 10.1001/jamanetworkopen.2021.35027

**Published:** 2021-10-22

In the Original Investigation titled “Mode of Obstetric Delivery in Kidney and Liver Transplant Recipients and Associated Maternal, Neonatal, and Graft Morbidity During 5 Decades of Clinical Practice,”^1^ published October 4, 2021, there was a space in the first name of Serban Constantinescu, MD, PhD, in the byline that should not have appeared there. This article has been corrected.
