# Supplementary material for: Mode of Obstetric Delivery in Kidney and Liver Transplant Recipients and Associated Maternal, Neonatal, and Graft Morbidity During 5 Decades of Clinical Practice
Source: JAMA Netw Open. 2021 Oct 4;4(10):e2127378. doi: 10.1001/jamanetworkopen.2021.27378 (PMC8491100; doi:10.1001/jamanetworkopen.2021.27378)
Supplement: Supplement. — eTable 1. Additional maternal characteristics according to mode of delivery among kidney and liver transplant recipients eTable 2. Primary indication for scheduled cesarean delivery (SCD) and cesarean delivery after trial of labor (TOL-CD) for pregnancies of kidney and liver transplant recipients eTable 3. Maternal, graft, and neonatal outcomes by mode of delivery among kidney and liver transplant recipients eTable 4. Individual indicators of severe maternal morbidity according to the Centers for Disease Control and Prevention eTable 5. Individual indicators of neonatal composite morbidity according to the NIH/NICHD [file jamanetwopen-e2127378-s001.pdf]

Supplementary Online Content

Yin O, Kallapur A, Coscia L, et al. Mode of obstetric delivery in kidney and liver transplant recipients and associated maternal, neonatal, and graft morbidity during 5 decades of clinical practice. *JAMA Netw Open*. 2021;4(10):e2127378. doi:10.1001/jamanetworkopen.2021.27378

eTable 1. Additional maternal characteristics according to mode of delivery among kidney and liver transplant recipients
eTable 2. Primary indication for scheduled cesarean delivery (SCD) and cesarean after trial of labor (TOL-CD) for pregnancies of kidney and liver transplant recipients
eTable 3. Maternal, graft, and neonatal outcomes by mode of delivery among kidney and liver transplant recipients
eTable 4. Individual indicators of severe maternal morbidity according to the Centers for Disease Control and Prevention
eTable 5. Individual indicators of neonatal composite morbidity according to the NIH/NICHD

This supplementary material has been provided by the authors to give readers additional information about their work.

© 2021 Yin O et al. *JAMA Network Open*.

eTable 1: Additional maternal characteristics according to mode of delivery among kidney and liver transplant recipients

	Kidney (n = 1435)			Liver (n = 430)		
		Trial of Labor			Trial of Labor	
**Maternal Outcome No. (%)**	**SCD (n = 459)**	**TOL-CD (n = 282)**	**TOL-VD (n = 694)**	**SCD (n = 105)**	**TOL-CD (n = 73)**	**TOL-VD (n = 252)**
Repeat transplant, No. (%)	76 (16.6)	52 (18.4)	91 (13.1)	8 (7.6)	10 (13.7)	16 (6.3)
BMI, No. (%), kg/m^2 [a, b]						
Underweight (<18.5)	8 (2.5)	9 (4.7)	26 (5.3)	2 (2.3)	1 (1.8)	12 (5.8)
Normal (18.5-24.9)	186 (56.9)	84 (44.0)	288 (58.4)	52 (58.4)	32 (57.1)	131 (63.0)
Overweight (25-29.9)	67 (20.5)	45 (23.6)	116 (23.5)	16 (18.0)	14 (25.0)	49 (23.6)
Obese (>30)	66 (20.2)	53 (27.8)	63 (12.8)	19 (21.4)	9 (16.1)	16 (7.7)
Census Region or Country, No. (%)						
Canada	10 (2.2)	7 (2.5)	23 (3.3)	3 (2.9)	4 (5.5)	6 (2.4)
Midwest	114 (24.8)	73 (25.9)	182 (26.2)	16 (15.2)	11 (15.1)	57 (22.6)
Northeast	99 (21.6)	71 (25.2)	154 (22.2)	23 (21.9)	18 (24.7)	44 (17.5)
South	100 (21.8)	69 (24.5)	152 (21.9)	32 (30.5)	17 (23.3)	53 (21.0)
West	67 (14.6)	31 (11.0)	109 (15.7)	18 (17.1)	15 (20.5)	53 (21.0)
Other	7 (1.5)	2 (0.7)	6 (0.9)	1 (1.0)	0 (0)	2 (0.8)
Unknown	62 (13.5)	29 (10.2)	68 (9.8)	12 (11.4)	8 (11.0)	37 (14.7)
Anemia or thrombo-cytopenia, No. (%)	44 (9.6)	22 (7.8)	60 (8.7)	6 (5.7)	4 (5.5)	32 (12.7)
Infections antepartum, No. (%)	93 (20.3)	66 (23.4)	145 (20.9)	21 (0.20)	15 (20.5)	64 (25.4)
Antepartum admission, No. (%)	33 (7.2)	24 (8.5)	40 (5.8)	13 (12.4)	6 (8.2)	14 (5.6)
PPROM/PTL[c], No. (%)	28 (6.1)	15 (5.3)	54 (7.8)	28 (6.1)	15 (5.3)	54 (7.8)

© 2021 Yin O et al. *JAMA Network Open*.

Antepartum severe maternal morbidity, No. (%)	7 (1.5)	2 (0.7)	4 (0.6)	3 (2.9)	0 (0)	5 (2.0)
Aspirin, No. (%)	33 (7.2)	29 (10.3)	43 (6.2)	4 (3.8)	7 (9.6)	18 (7.1)
Anticoagulation, No. (%)	13 (2.8)	8 (2.8)	11 (1.6)	3 (2.9)	4 (5.5)	9 (3.6)
Antepartum bleeding, No. (%)	4 (0.9)	5 (1.8)	12 (1.7)	5 (4.8)	3 (4.1)	5 (2.0)

a. p≤0.05
b. Variables with > 5% missing include:
BMI: 424 (29.5%) kidney, 77 (17.9%) liver
c. Preterm prelabor rupture of membranes (PPROM)/preterm labor (PTL)

eTable 2: Primary indication for scheduled cesarean delivery (SCD) and cesarean after trial of labor (TOL-CD) for pregnancies of kidney and liver transplant recipients

	**Kidney**	**Liver**
**Scheduled CD (SCD) No. (%)**	**(n=459)**	**(n=105)**
Non-medically indicated	124 (27.0)	21 (20.0)
Repeat CD	105 (22.9)	29 (27.6)
Hypertensive disorder	98 (21.4)	16 (15.2)
Malpresentation	53 (11.5)	19 (18.1)
Growth restriction/oligohydramnios	9 (2.0)	6 (5.7)
Abnormal antenatal testing	14 (3.1)	1 (1.0)
Abnormal placentation	8 (1.7)	4 (3.8)
Other[a]	19 (4.1)	7 (6.7)
Unknown	29 (6.3)	2 (1.9)
**Trial of labor to cesarean (TOL-CD) No. (%)**	**(n=282)**	**(n=73)**
Failure to progress	128 (45.4)	31 (42.5)
Non-reassuring fetal heart tracing	99 (35.1)	28 (38.4)
Hypertensive disorder	16 (5.7)	3 (4.1)
Non-medically indicated	5 (1.8)	1 (1.4)
Other[b]	8 (2.8)	3 (4.1)
Unknown	26 (9.2)	7 (9.6)

a. ‘Other’ includes ‘Abruption', 'Anesthesia', 'Hepatitis C, 'Herpes', 'Osteoporosis', 'Prior uterine scar’ and 'Worsening renal function’
b. ‘Other’ includes ‘Abruption’, ‘Fetal growth restriction’, ‘Malpresentation’, ‘Anesthesia’, and ‘Worsening renal function’

© 2021 Yin O et al. *JAMA Network Open*.

eTable 3: Maternal, graft, and neonatal outcomes by mode of delivery among kidney and liver transplant recipients

	Kidney (n = 1435)			Liver (n = 430)		
		Trial of Labor			Trial of Labor	
**Maternal Outcome No. (%)**	**SCD (n = 459)**	**TOL-CD (n = 282)**	**TOL-VD (n = 694)**	**SCD (n = 105)**	**TOL-CD (n = 73)**	**TOL-VD (n = 252)**
Postpartum hemorrhage	7 (1.5)	6 (2.1)	18 (2.6)	1 (1.0)	2 (2.7)	10 (4.0)
Intraamniotic infection	3 (0.7)	3 (1.1)	3 (0.4)	2 (1.9)	1 (1.4)	2 (0.8)
Surgical site infection	6 (1.3)	4 (1.4)	2 (0.3)	4 (3.8)	3 (4.1)	0 (0)
Postpartum Readmission	3 (0.7)	3 (1.1)	5 (0.7)	1 (1)	0 (0)	1 (0.4)
Severe maternal morbidity	13 (2.8)	12 (4.3)	18 (2.6)	2 (1.9)	3 (4.1)	7 (2.8)
Graft loss within 2 years	29 (6.3)	18 (6.4)	33 (4.8)	2 (1.9)	1 (1.4)	4 (1.6)
	**Kidney (n = 1486)**			**Liver (n = 440)**		
		**Trial of Labor**			**Trial of Labor**	
**Neonatal Outcome No. (%)**	**SCD (n = 494)**	**TOL-CD (n = 285)**	**TOL-VD (n = 707)**	**SCD (n = 113)**	**TOL-CD (n = 73)**	**TOL-VD (n = 254)**
Apgar score at 1 minute <7 [a]	24 (18.5)	27 (25.7)	43 (19.7)	7 (35.0)	5 (35.7)	7 (15.6)
Apgar score at 5 minutes <7 [a]	8 (6.2)	9 (8.6)	5 (2.3)	4 (20.0)	2 (15.4)	0 (0)
NICU admission	137 (27.7)	59 (20.7)	102 (14.4)	33 (29.2)	17 (23.3)	37 (14.6)
NICU length of stay, days, median (IQR)	26 (10, 42)	14 (7, 27)	11 (7, 21)	21 (4.5, 46)	12 (4, 17.5)	14 (5, 35)
Neonatal composite morbidity [a]	103 (20.9)	31 (10.9)	53 (7.5)	18 (15.9)	7 (9.6)	17 (6.7)

a. Variables with > 5% missing include:

Apgar 1 minute: 1033 (69.5%) kidney, 361 (82.0%) liver

Apgar 5 minute: 1033 (69.5%) kidney, 363 (82.5%) liver

© 2021 Yin O et al. *JAMA Network Open*.

eTable 4: Individual indicators of severe maternal morbidity according to the Centers for Disease Control and Prevention

	Kidney (n = 1435)			Liver (n = 430)		
		Trial of Labor			Trial of Labor	
**Indicator No. (%)** [a]	**SCD (n = 459)**	**TOL-CD (n = 282)**	**TOL-VD (n = 694)**	**SCD (n = 105)**	**TOL-CD (n = 73)**	**TOL-VD (n = 252)**
Acute myocardial infarction	0 (0%)	1 (0.4%)	1 (0.1%)	0 (0%)	0 (0%)	0 (0%)
Aneurysm	0 (0%)	0 (0%)	0 (0%)	0 (0%)	0 (0%)	1 (0.4%)
Acute renal failure	1 (0.2%)	1 (0.4%)	0 (0%)	0 (0%)	0 (0%)	0 (0%)
Conversion of cardiac rhythm	0 (0%)	1 (0.4%)	0 (0%)	0 (0%)	0 (0%)	0 (0%)
Disseminated intravascular coagulation	0 (0%)	0 (0%)	1 (0.1%)	0 (0%)	0 (0%)	0 (0%)
Eclampsia	1 (0.2%)	2 (0.7%)	1 (0.1%)	0 (0%)	0 (0%)	0 (0%)
Pulmonary edema/acute heart failure	0 (0%)	1 (0.4%)	0 (0%)	1 (1.0%)	0 (0%)	0 (0%)
Ventilation	1 (0.2%)	0 (0%)	0 (0%)	0 (0%)	0 (0%)	0 (0%)
Sepsis	3 (0.7%)	2 (0.7%)	5 (0.7%)	0 (0%)	1 (1.4%)	1 (0.4%)
Air and thrombotic embolism[a]	1 (0.2%)	0 (0%)	2 (0.3%)	0 (0%)	0 (0%)	0 (0%)
Blood products transfusion	5 (1.1%)	3 (1.1%)	8 (1.2%)	1 (1.0%)	2 (2.7%)	5 (2.0%)
Hysterectomy	1 (0.2%)	1 (0.4%)	0 (0%)	0 (0%)	0 (0%)	0 (0%)

a. The following severe maternal morbidity indicators did not occur so were omitted from the table: acute respiratory distress syndrome, amniotic fluid embolism, cardiac arrest/ventricular fibrillation, heart failure/arrest during surgery or procedure, puerperal cerebrovascular disorders, severe anesthesia complications, shock, sickle cell disease with crisis, and temporary tracheostomy.
b. Only thrombotic embolism occurred in this study

© 2021 Yin O et al. *JAMA Network Open*.

eTable 5: Individual indicators of neonatal composite morbidity according to the NIH/NICHD

	Kidney (n = 1486)			Liver (n = 440)		
		Trial of Labor			Trial of Labor	
**Neonatal Outcome No. (%)**	**SCD (n = 494)**	**TOL-CD (n = 285)**	**TOL-VD (n = 707)**	**SCD (n = 113)**	**TOL-CD (n = 73)**	**TOL-VD (n = 254)**
Respiratory distress syndrome	64 (13.0)	19 (6.7)	22 (3.1)	6 (5.3)	5 (6.8)	5 (2.0)
Mechanical ventilation within 24 hours	3 (0.6)	1 (0.4)	2 (0.3)	3 (2.7)	0 (0)	0 (0)
Cardiopulmonary resuscitation	1 (0.2)	0 (0)	0 (0)	0 (0)	0 (0)	0 (0)
Persistent pulmonary hypertension	0 (0)	0 (0)	0 (0)	0 (0)	0 (0)	0 (0)
Bronchopulmonary dysplasia	11 (2.2)	0 (0)	4 (0.6)	3 (2.7)	0 (0)	2 (0.8)
Sepsis including necrotizing entero-colitis	9 (1.8)	6 (2.1)	11 (1.6)	3 (2.7)	0 (0)	6 (2.4)
Birth trauma	0 (0)	0 (0)	1 (0.1)	0 (0)	0 (0)	1 (0.4)
Seizure	0 (0)	1 (0.4)	0 (0)	0 (0)	0 (0)	0 (0)
Intraventricular hemorrhage	3 (0.6)	2 (0.7)	2 (0.3)	2 (1.8)	0 (0)	1 (0.4)
Hypoxic ischemic encephalopathy	3 (0.6)	1 (0.4)	1 (0.1)	1 (0.9)	1 (1.4)	0 (0)
Perinatal death	9 (1.8)	1 (0.4)	10 (1.4)	0 (0)	1 (1.4)	2 (0.8)

© 2021 Yin O et al. *JAMA Network Open*.
